# The Role of Type 4 Phosphodiesterases in Generating Microdomains of cAMP: Large Scale Stochastic Simulations

**DOI:** 10.1371/journal.pone.0011725

**Published:** 2010-07-22

**Authors:** Rodrigo F. Oliveira, Anna Terrin, Giulietta Di Benedetto, Robert C. Cannon, Wonryull Koh, MyungSook Kim, Manuela Zaccolo, Kim T. Blackwell

**Affiliations:** 1 The Krasnow Institute for Advanced Study, George Mason University, Fairfax, Virginia, United States of America; 2 Faculty of Biomedical and Life Sciences, University of Glasgow, Glasgow, Scotland, United Kingdom; 3 Venetian Institute of Molecular Medicine, Padova, Veneto, Italy; 4 Textensor Limited, Edinburgh, Scotland, United Kingdom; Mount Sinai School of Medicine, United States of America

## Abstract

Cyclic AMP (cAMP) and its main effector Protein Kinase A (PKA) are critical for several aspects of neuronal function including synaptic plasticity. Specificity of synaptic plasticity requires that cAMP activates PKA in a highly localized manner despite the speed with which cAMP diffuses. Two mechanisms have been proposed to produce localized elevations in cAMP, known as microdomains: impeded diffusion, and high phosphodiesterase (PDE) activity. This paper investigates the mechanism of localized cAMP signaling using a computational model of the biochemical network in the HEK293 cell, which is a subset of pathways involved in PKA-dependent synaptic plasticity. This biochemical network includes cAMP production, PKA activation, and cAMP degradation by PDE activity. The model is implemented in NeuroRD: novel, computationally efficient, stochastic reaction-diffusion software, and is constrained by intracellular cAMP dynamics that were determined experimentally by real-time imaging using an Epac-based FRET sensor (H30). The model reproduces the high concentration cAMP microdomain in the submembrane region, distinct from the lower concentration of cAMP in the cytosol. Simulations further demonstrate that generation of the cAMP microdomain requires a pool of PDE4D anchored in the cytosol and also requires PKA-mediated phosphorylation of PDE4D which increases its activity. The microdomain does not require impeded diffusion of cAMP, confirming that barriers are not required for microdomains. The simulations reported here further demonstrate the utility of the new stochastic reaction-diffusion algorithm for exploring signaling pathways in spatially complex structures such as neurons.

## Introduction

cAMP is an important second messenger molecule responsible for the regulation of many aspects of neuronal function. For instance, cAMP signaling plays a critical role in the late phase of LTP through its main effector PKA [1] and in psychiatric diseases such as schizophrenia, in which the disruption of the interaction between DISC-1 (a scaffold protein) and PDE activity [2] produces altered cAMP activity. In cardiac cells cAMP is a key regulator of the excitation-contraction cycle through the control of intracellular calcium concentration mediated by PKA phosphorylation of a number of targets including L-type calcium channels [3]. cAMP also regulates gene transcription through cAMP-response element binding protein (CREB), a transcription factor that regulates expression of genes implicated in neuroplasticity and cognition [4], [5]. Accomplishment of these various functions in a specific manner requires a highly localized PKA activity (for instance, at the nucleus in gene regulation and at the subplasma membrane in channel phosphorylation). This localized PKA activity seems incompatible with the highly diffusible nature of the cAMP molecule. To achieve selective activation, PKA is localized to defined compartments within the neuron by binding to A-Kinase-Anchoring-Proteins [6] and cAMP is compartmentalized in different cellular microdomains [7]–[9]. How these microdomains are maintained is an open question with important implications for information processing in signalling pathways.

The inhomogeneous cAMP concentration in different cellular subregions results from the interplay of three processes: 1) synthesis by adenylate cyclase (AC) that is activated by G protein-coupled receptors (GPCRs) on the plasma membrane, 2) degradation by phosphodiesterases (PDEs) and, 3) diffusion. One potential mechanism for producing cAMP microdomains is a physical barrier impeding diffusion away from its production site [10]–[13]. Another mechanism is colocalization of cAMP production with its target molecules while simultaneously having high levels of PDEs. The result of this arrangement would be a high local concentration of cAMP but a low cytosolic concentration preventing diffuse activation of cAMP targets. Recent evidence has been accumulating in favor of an active role for phosphodiesterases in regulating cAMP concentration [3], [14]–[16].

The investigation of cAMP microdomains requires the use of techniques with high temporal and spatial resolution. Fluorescence resonance energy transfer (FRET) is an invaluable imaging technique for investigating the dynamics of molecular interactions in living cells [17]–[21]. The principle of FRET is the ability of a high energy fluorophore (donor) to transfer energy to a lower energy fluorophore (acceptor) when the two are within 1–10 nm [21]. Thus, a change in molecule conformation upon cAMP binding, such as occurs with the Epac-based H30 sensor, produces a change in fluorescence that is detectable in real-time with high spatial resolution in living cells [16].

A complementary approach to investigating cAMP microdomains uses computational modeling techniques (e.g. [22]). Although FRET imaging provides invaluable evidence on the location and relative changes in second messenger concentrations in living cells, the absolute cAMP concentration must be inferred using an additional, experimental FRET calibration. In addition, cells contain a diverse and complex signaling network with many molecules that may influence cAMP microdomains. Computational simulations that are constrained by experimental data play a distinctive role through evaluating the robustness of a hypothesis, explicating hidden assumptions in a conceptual model, or testing causal relationships and hypotheses. The small size of microdomains implies that there are only a few molecules reacting and diffusing, and requires stochastic algorithms for accurate simulation of reactions and diffusion. Concurrently, the large numbers of molecules in a cell requires computationally efficient stochastic algorithms.

This paper uses computational modeling to explore the molecular mechanisms responsible for cAMP microdomains. The model is implemented using novel, stochastic reaction-diffusion software, *NeuroRD*, developed for efficient stochastic modeling of large biochemical networks in relatively large volumes such as a neuronal dendrite with multiple spines. This mesoscopic algorithm blends the stochastic diffusion algorithm of Blackwell [23] with the tau-leap stochastic reaction algorithm of Gillespie [24]. The validity of the algorithm is demonstrated by comparison with a previously published software [25]. The utility of the algorithm is demonstrated by investigating the role of PDEs and PKA in producing cAMP microdomains. The model not only simulates cAMP production, PKA activation and compartmentalized PDE activity in a HEK293 cell, but also includes the unimolecular Epac-based FRET sensor H30, in order to compare simulated cAMP dynamics to that measured experimentally using H30 [16].

## Materials and Methods

### Model Description

**Figure 1:**
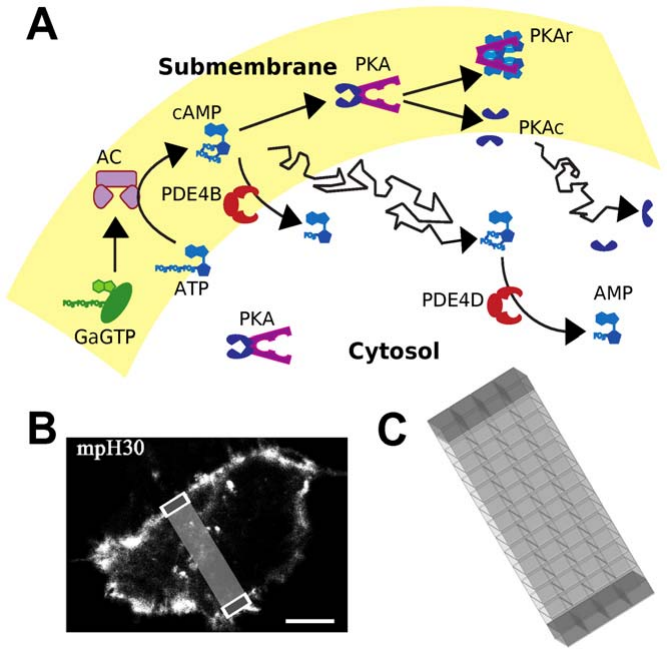
Schematic representation of the biochemical signaling pathway modeled. (A) G_α_GTP binds to and activates adenylate cyclase, which then produces cAMP from ATP. cAMP activates PKA, a heterotetramer with two regulatory and two catalytic subunits. After binding 4 molecules of cAMP, the two catalytic subunits (PKAc) dissociate from the regulatory subunit dimer (PKAr) and become active [26], [27]. cAMP is degraded by phosphodiesterase, type 4B (PDE4B) and type 4D (PDE4D). AC, G_α_GTP, PKA and PDE4B are anchored at the submembrane while PKA and PDE4D are distributed throughout the cytosol. cAMP, ATP, AMP and PKAc freely diffuse. (B) Confocal image showing the localization of the membrane-targeted version of the unimolecular Epac-based sensor for cAMP (mpH30) in HEK293 cells. Confocal images were acquired 24 hours after transfection by using the broadband confocal Leica TCS SP5 system (Leica Microsystems) and a HCX PL APO 63x1.4NA oil-immersion objective (scale bar 10 µm). The representation superimposed on the micrograph corresponds to the grid in C. (C) Schematic representation of the spatial structure of the HEK293 cell model, light gray compartments correspond to the cytosol while dark gray compartments correspond to the submembrane region in a slice of the 3-dimensional cell.

A computational model of cAMP production and degradation is employed to explore the generation of cAMP microdomains, which are important for synaptic specificity. Because this set of cAMP signaling pathways is widespread, we explore mechanisms underlying cAMP microdomains in a HEK293 cell (Fig. 1A), for which experimental measures of these microdomains provide model constraints. In this model, cAMP is produced from ATP by adenylate cyclase, which is activated by G_α_GTP binding. ATP is regenerated by a first order reaction AMP→ATP to prevent depletion. cAMP activates PKA, a heterotetramer with two regulatory and two catalytic subunits. After binding 4 molecules of cAMP, the two catalytic subunits (PKAc) dissociate from the regulatory subunit dimer (PKAr) and become active [26], [27]. As described below, to compare with FRET imaging data, the model also includes the Epac-based FRET sensor H30, which binds a single cAMP molecule.

PDEs are responsible for cAMP degradation, converting it into AMP. The prevalent PDE activity in HEK293 cells is provided by PDE4 isozymes. In particular, PDE4B is responsible for 30% of the total PDE4 activity and is located in the submembrane region, and PDE4D is responsible for 60% of the PDE4 activity and is located in the cytosol [15], [16]. In addition, these PDE4 isoforms are phosphorylated by PKA with a resulting increase in activity [28], [29].

Rate constants for reactions were constrained with published biochemical rate constants as listed in Table 1. The diffusion constants (Table 2) were adjusted using the equation suggested by Young et al. [30]:
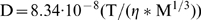
(1)where the diffusion coefficient *D* was in cm^2^·s^−1^, *T* was temperature in K, the solution viscosity *η* was in cP, and molecular weight *M* was in g·mol^−1^. Making the diffusion constant inversely proportional to molecular weight was based on the assumption that the Stoke's radius of a molecule was approximated by the molecular weight. The diffusion constant was calculated for all diffusible molecules included in the model (cAMP, ATP, AMP and PKA catalytic subunit) using a cytosolic viscosity value (1.2 cP) from Fushimi and Verkman [31]. The resulting diffusion constants agreed with available experimentally measured values (Table 2).

**Table 1:** Reactions and rate constants of HEK293 cell biochemical network.

Reaction	K_f_ (nM^−1^ sec^−1^)	K_b_ (sec^−1^)	K_cat_ (sec^−1^)	Reference
G_α_GTP+AC↔E	3.85×10^−2^	10		[57]
E+ATP↔EATP	1.28×10^−4^	2.61×10^−1^		Adapted from [58]
EATP↔E+cAMP	28.46	2.59×10^−4^		
2(cAMP)+PKA↔PKAr_2_c_2_cAMP_2_	8.70×10^−5^	0.02		[26], [27]
2(cAMP)+PKAr_2_c_2_cAMP_2_↔PKAr_2_c_2_cAMP_4_	1.15×10^−4^	0.2		
PKAr_2_c_2_cAMP_4_↔PKAr_2_cAMP_4_+2(PKAc)	0.016	0.0017		
cAMP+PDE4B↔PDE4BcAMP→AMP+PDE4B	0.03	77.44	19.36	[59]
PKAc+PDE4B↔PKAcPDE4B→pPDE4B+PKAc	3.375×10^−4^	4.08×10^−1^	4.17×10^−1^	Estimated
PKAc+PDE4BcAMP↔PKAcPDE4BcAMP→pPDE4BcAMP+PKAc	3.375×10^−4^	4.08×10^−1^	4.17×10^−1^	Estimated
cAMP+pPDE4B↔pPDE4BcAMP→AMP+pPDE4B	0.03	77.44	27.10	[29]
pPDE4B↔PDE4B	10.88×10^−3^			Estimated
cAMP+PDE4D↔PDE4DcAMP→AMP+PDE4D	0.012	58.82	14.70	[59]
PKAc+PDE4D↔PKAcPDE4D→pPDE4D+PKAc	6.25×10^−4^	5.44×10^−3^	5.56×10^−3^	Estimated
PKAc+PDE4DcAMP↔PKAcPDE4DcAMP→pPDE4DcAMP+PKAc	3.375×10^−4^	4.08×10^−1^	4.17×10^−1^	Estimated
cAMP+pPDE4D↔pPDE4DcAMP→AMP+pPDE4D	0.024	58.82	92.58	[29], [60]
pPDE4D↔PDE4D	10.88×10^−3^			Estimated
H30+cAMP↔H30cAMP	12.21×10^−7^	2.17×10^−2^		Constrained by data (see Methods)
AMP→ATP	10.85×10^−2^			Estimated

**Table 2:** Initial concentrations for molecules in the model.

Specie	Ic (S)	Ic (C)	K_diff_
G_α_GTP	3.059	3.509	NA
AC	2.7×10^4^	0	0
E	362.8	0	0
ATP	1.904×10^6^	1.90×10^6^	255.34/[61]: 248 µm^2^/s
EATP	3073	0	0
cAMP	1134	541.8	294.91/[62]: 330 µm^2^/s, [63]: 270 µm^2^/s, [64]: 700 µm^2^/s
AMP	1074	1073	289.72
PKA	292.6	173.6	0
PKAr_2_c_2_cAMP_2_	1523	370.5	0
PKAr_2_c_2_cAMP_4_	984.3	115.6	0
PKAc	23.66	23.61	59.54
PKAr	408.6	47.59	0
PDE4B	292.2	0	0
PDE4BcAMP	101.3	0	0
pPDE4B	65.17	0	0
pPDE4BcAMP	183.7	0	0
PKAcPDE4B	4.551	0	0
PKAcPDE4BcAMP	1.691		
PDE4D	0	2196	0
PDE4DcAMP	0	191.5	0
pPDE4D	0	1219	0
pPDE4DcAMP	0	108.1	0
PKAcPDE4D	0	43.84	0
PKAcPDE4DcAMP		3.755	

Concentrations (in nM) for submembrane (S) and cytosolic (C) compartments and diffusion constants (K_diff_ in µm^2^/s) are calculated based on molecular weight of molecular species in the HEK293 cell model (0 denotes non-diffusible molecule and NA denotes NOT APLICABLE). Initial concentrations reported in this table are extracted from output of simulations without expression of biosensor. References in K_diff_ column allow comparison between experimentally measured and calculated (eq. 1) diffusion constants.

*Spatial Structure*. The spatial structure used to investigate the cAMP concentration microdomain in the HEK293 cell computational model included 60 biochemical subvolumes with equal volumes (0.93×0.93×0.5 µm) aligned in a 2-dimensional grid (4×15 subvolumes, with reflective boundary conditions). This grid was a 2-dimensional slice of the 3-dimensional cell (Fig. 1B), analogous to the slice of the cell in focus in the imaging experiments.. This structure allowed for the inclusion of two main subregions with distinctive cAMP concentrations: the submembrane region (set of subvolumes located on the margins of the system) and the cytosol (Fig. 1C, submembrane in dark grey and cytosol in light grey). AC, G_α_GTP, PKA and PDE4B were anchored in the submembrane subvolumes; the cytosolic subvolumes contained anchored PDE4D and a smaller quantity of PKA. All subvolumes contained the diffusible species cAMP, ATP, AMP and PKA catalytic subunit (Fig. 1A). Simulations were repeated using smaller subvolumes (with no change in total simulated volume) to demonstrate that the simulation results were not dependent on subvolume size (see details in Robustness to Parameter Variation and Fig. S5).

Since the submembrane region has a small volume, the concentration of the submembrane anchored signaling molecules correspond to a small number of molecules. Therefore reactions occur stochastically (randomly) and the variability observed with infrequent reactions cannot always be averaged out. Similarly, diffusion of molecules to and from small volume regions also occur probabilistically and an average description is often insufficient. Furthermore, using a biochemical model of a spine, Bhalla et al. demonstrate that the computational properties (i.e. bistability, threshold) of synaptic signaling pathways that are exhibited in deterministic systems are not necessarily maintained in stochastic systems [32]. To account for this stochastic behavior of reactions and diffusion, the model is implemented using the *NeuroRD* software to investigate cAMP microdomains in the HEK293 cell model.

*Stimulation*. To simulate prostaglandin (PGE) receptor stimulation (as performed in [16]) G_α_GTP was injected into the cell for 2 s, at a constant rate of 50 particles/ms. This simplification (excluding details of GPCR activation and desensitization) was used, rather than the dynamic production and degradation of G_α_GTP as occurred in experiments, to limit the scope of the model to mechanisms underlying microdomains. For simulations with reduced PDE activity, G_α_GTP injection was reduced to prevent non-physiological cAMP concentration and to avoid FRET saturation (again consistent with experiments).

### NeuroRD Software

*NeuroRD* is a novel software tool for simulating reaction diffusion systems taking into account the stochasticity of molecular interactions and movement. It uses a mesoscopic Monte Carlo approach to follow populations of molecules in a tesselated space to avoid the computational burden of simulating molecules individually. The software merges the tau-leap algorithm of Gillespise [24], which allows multiple reaction events at each time step, with the diffusion algorithm of Blackwell [23], which allows multiple diffusion events at each time step. Further efficiency is achieved by the use of pre-computed lookup tables that store cumulative binomial probabilities of the number of molecules diffusing or reacting. Though approximate, it retains sufficient accuracy to allow simulation of large and complex spatial structures containing various interacting diffusible and non-diffusible molecules. The specific purpose of this algorithm is to allow simulations that lie between deterministic methods, which have large volume capabilities, and microscopic simulators, such as MCell [33] and Smoldyn [25], which simulate molecules diffusing without tessellating space and simulate molecule reactions based on proximity (approximate collisions). NeuroRD is currently not able to simulate lateral diffusion in membranes, or molecular crowding for which the more detailed simulators may be required. NeuroRD is written in Java, so that it can be run on most common platforms, and is freely available for download (http://krasnow.gmu.edu/CENlab/).

The software operates as a linear processing pipeline, taking a declarative specification of the model and simulation parameters as the input and generating files of simulation data as the output. There is no graphical user interface and it does not support a scripting language. Instead, models are specified using a set of user friendly xml files, in order to separate description of the model from description of the simulation itself [34]. The reactions and diffusion constants are specified in one file. The morphology is specified in a separate file, to facilitate the investigation of the role of morphology, and to allow different morphologies to be investigated with different signaling pathways and vice versa. Initial conditions are specified in a third file, to facilitate evaluating robustness of the results to molecule quantities. Stimulation (influx of molecules to initiate reactions) and desired output are each specified in additional files. All of these xml files are specified in a top level xml file, which contains additional details about the simulation itself (i.e. integration time step, random seed). The results, in the form of the number of particles of each type in each element at each timestep, are stored to files for processing once the simulation has completed. Details on how to implement models using NeuroRD are explained in the HEK293 xml files available for download from the author's website, and in the “readme” file accompanying the software.

The first stage of processing is to tesselate the spatial structure with cuboid elements. The model specification allows the structure to be expressed in a manner similar to that used in MorphML [35] or CVAPP [36] as a set of connected points, each with a 3D position and radius. This structure is converted to the nearest equivalent set of cuboids in either 2 or 3 dimensions. Given adjacent elements with a contact area A between them and center-to-center distance *l*, the probability, *pm*, of a molecule moving between them in time Δt is:
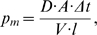
(2)where *D* is the diffusion constant for the species in question, *V* is the volume of the element from which it is diffusing.

The probability *p_r_* of a reaction event taking place between populations A and B is:
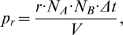
(3)where *N_A_* and *N_B_* are the number of molecules of the two reactants and *r* is the reaction rate. Note that the right hand side of equation (2) is dependent only on the geometry of the spatial discretization. It does not change during the simulation, so these quantities (and their logarithms, for computational efficiency) can be computed once at the start of the simulation.

Given the reaction and diffusion probabilities, each step of the calculation involves generating the numbers of particles diffusing across each possible boundary, the number of reactions taking place, and updating the number of particles of each type in each element accordingly (Fig. S1A). The reaction and diffusion steps are done successively, so in effect they are offset by half a timestep as in a leap-frog method, but for each of the two processes all events are generated before any populations are updated so as to avoid systematic errors arising from the sequence of operations.

The most time consuming part of the calculation is generating the number of particles making a particular transition (either reaction or diffusion), which depends on the size of the source population, N, and the probability, p, of a single particle making that transition (Fig. S1B). For large populations, Gaussian (Np>20) or Poisson distributions (Np<20) are used. For smaller populations, up to 120 particles, the number of events is generated from a single random number by interpolation in precomputed lookup tables [23]. Tables are computed on a logarithmic grid of probabilities between 10^−8^ and 0.5. For each probability, p, and population size N, the table contains the cumulative probability, c, of k or fewer particles making a transition:
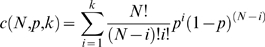
(4)where p is either p_r_ for reactions or p_m_ for diffusion. For an event for which the probability is present in the table (which in general will not be the case, but can be arranged, for example for a small number of diffusing species on a regular grid where only a few different probabilities occur), generating a corresponding number of transitions involves generating a uniform random number, u, and walking through the table to find the corresponding k such that
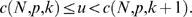
(5)


For probabilities not directly present in the table, the same approach is used but a linear interpolation is performed between the adjacent rows.

Once the number of diffusing particles is calculated, the destination of the particles is determined (Fig S1C). NeuroRD supports two strategies for determining the destination when there are multiple possible destinations, such as different boundaries to cross, for a given particle. It can generate the numbers of particles taking each route independently or it can generate the total number of particles taking any of the routes and then allocate particles from this total to the different routes according to their relative probabilities. The latter method is used when the number of particles taking any route is small (less than 4 times the number of adjacent subvolumes), to avoid generating negative numbers of particles when the number of source particles is small.

All simulations described in this paper were performed using a computer cluster composed of nodes with Intel(R) Xeon(R) 2.66GHz processors (X5355, 4096 KB cache) and 8 GB (8048408 kB) of RAM memory. The algorithm was not parallelized and each simulation was performed on a single node independently. Unless otherwise noted, a simulation timestep of 0.1 ms was used.

### FRET sensor equation

The original experiments [16] utilize a sensor with cyan as the donor's wavelength and yellow as the acceptor's wavelength. When H30 is free, a fraction, β, of the donor's emission is transferred to the acceptor fluorophore, which emission is detected in the yellow channel; the remaining fraction (1−β) of the donor's emission is detected in the cyan channel. δ and γ represent overlap of emission and excitation spectra, respectively. γ represents overlap at excitation spectra, i.e. the cyan excitation wavelength (430 nm) partially excites the yellow fluorophore [19] (for a review see [37]). δ represents donor emission into the acceptor channel (donor signal bleed through). Experiments report the FRET ratio, which is volume and concentration independent and is the ratio of cyan (acceptor's) signal to the yellow (donor's) signal.

**Figure 2:**
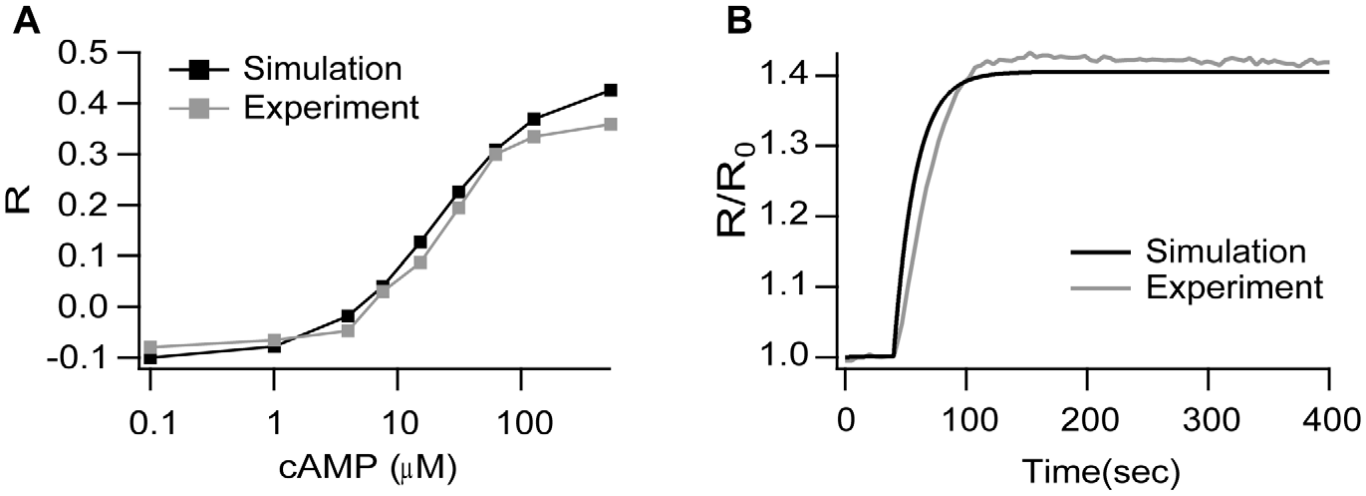
FRET signal as a function of cAMP concentration. (A) Steady state dose-response simulation (black) and experimental (gray) curves show excellent agreement. Ordinates are background-subtracted FRET emission ratio changes, ΔR, measured relative to the prestimulus ratio R(0). (B) Time course of simulated and experimental FRET signal shows excellent agreement. FRET ratio trace obtained by delivery of 30 µM cAMP to cell under whole-cell recording conditions. All experiments were performed in HeLa cells transfected with H30 (acquisition conditions: 1 frame/5 s). The microscope was equipped with a CCD camera (Sensicam QE; PCO), a software-controlled monochromator (Polychrome IV; TILL Photonics), and a beam-splitter optical device (Multispec Microimager; Optical Insights).

In order to precisely compare simulated results with FRET imaging data, a theoretical FRET signal was calculated from simulated concentrations of cAMP-bound-H30 and free H30 sensor, and included a FRET efficiency term and contamination terms due to overlap of the sensor emission and excitation spectra. Thus, the simulated FRET ratio, R, was the same as the experimental FRET ratio:
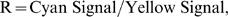
(6)with
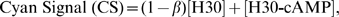
(7)

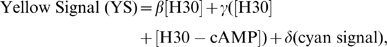
(8)and β = 0.35, γ = 0.12, δ = 0.67. When H30 was bound to cAMP there was no transfer of energy and all of the donor emission was detected as the cyan signal. β, γ, δ and H30 affinity for cAMP were obtained from experimental measurements and were adjusted slightly to yield better agreement with experimental calibration data (Fig. 2). R/R_0_ was calculated by dividing FRET ratio, R(t), by the initial FRET ratio, R(0), measured before stimulation was applied, as in original experiments. Simulation of cAMP dose-FRET response curves were constructed and compared to experiments (Fig. 2A). In addition, the time course of simulated H30 binding to cAMP, as measured by the FRET signal, also was compared to experimental results. Both FRET calibration curves showed good agreement (Fig. 2B), providing precise quantitative comparison with experimental results.

For each comparison of simulated FRET microdomains with experimental results, we performed two simulations. In one simulation H30 was included as a submembrane anchored protein, and the FRET was calculated from submembrane concentrations of bound and free H30 only. In the other simulation, H30 was included as a cytosolic protein, and FRET calculated from cytosolic concentrations. This approach was identical to experiments, in which the FRET sensor was expressed as a submembrane-bound molecule in one set of cell cultures, and was expressed as a cytosolic molecule in a different set of cell cultures. All simulations had the same number of H30 molecules: when included as a submembrane anchored protein, concentration was 1609 nM in a submembrane volume of 3.46 µm^3^; when included as a cytosolic protein H30 concentration was 266 nM in a cytosolic volume of 22.48 µm^3^. The FRET ratio was robust and largely independent of the concentration of the sensor.

### Experiments

#### Reagents

DME, Opti-MEM, FBS, L-glutamine, penicillin, trypsin/EDTA, PBS, and LipofectAMINE 2000 were purchased from Invitrogen. PGE1 was obtained from Sigma-Aldrich. FuGENE-6 transfection reagent was obtained from Roche.

#### Cell culture and transfection

Human embryonic kidney cells (HEK293) were grown in DME containing 10% FBS supplemented with 2 mM L-glutamine, 100 U/ml penicillin, and 100 µg/ml streptomycin in a humidified atmosphere containing 5% CO_2_. For transient expression of the Epac-based FRET sensor [16], cells were seeded onto 24-mm diameter round glass coverslips, and transfections were performed at 50–70% confluence with FuGENE-6 transfection reagent according to the manufacturer's instructions using 1–2 µg DNA per coverslip. Imaging experiments were performed 24–48 h after transfection with either H30, or mH30. For selective knockdown of PDE4B or PDE4D subfamilies, double-stranded 21-mer RNA duplexes (Dharmacon) targeted at regions of sequence that are unique to each of these subfamilies were used, as described previously [15].

#### Selective knockdown of PDE4 subfamilies

Each siRNA duplex was delivered into target cells via the reagent LipofectAMINE 2000 (Invitrogen). Specifically, 5 µl LipofectAMINE 2000 (1 mg/ml) was diluted in 100 µl Opti-MEM, and, separately, 125 pmol of each siRNA sample and 1 µg cAMP sensor DNA were diluted in 100 µl Opti-MEM. 200 µl siRNA–DNA transfection complexes were added to each well, and the plates were incubated for 3–4 h at 37°C (5% CO2). These complexes were then removed and replaced with DME. Imaging experiments were performed after 48 h.

#### FRET imaging

Cells were maintained in Hepes-buffered Ringer-modified saline containing 125 mM NaCl, 5 mM KCl, 1 mM Na3PO_4_, 1 mM MgS0_4_, 5.5 mM glucose, 1 mM CaCl_2_, and 20 mM Hepes, pH 7.5, at room temperature (20–22°C) and imaged on an inverted microscope (IX50; Olympus) with a 60× NA 1.4 oil immersion objective (Olympus). Images were acquired using custom-made software and processed using ImageJ (National Institutes of Health). FRET changes were measured as changes in the background- subtracted 480/545-nm fluorescence emission intensities on excitation at 430 nm and expressed as either R/R_0_, where R is the ratio at time t and R_0_ is the ratio at time = 0 s, or ΔR/R_0_, where ΔR = R−R_0_.

## Results

### Validation of software

**Figure 3:**
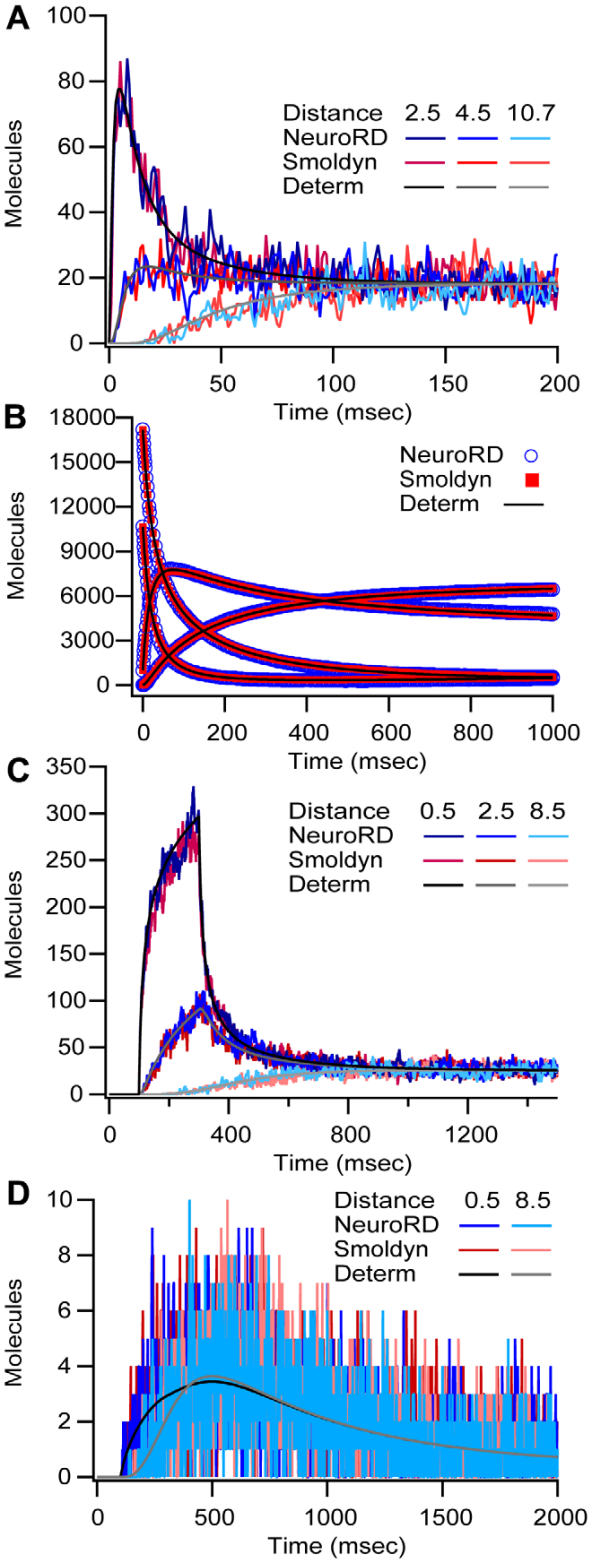
Validation of *NeuroRD*. Simulations show good agreement between NeuroRD, Smoldyn [25] and deterministic solutions (XPPAUT [38] or Chemesis [39]). (A) Validation of diffusion alone. Deterministic trace generated using Chemesis; (B) Validation of reactions alone. The deterministic trace is generated using XPPAUT; (C and D) Validation of reaction-diffusion. The deterministic trace is generated using Chemesis. In all panels *Distance* refers to the Euclidean distance in microns between center of source subvolume and center of other subvolumes. Panel C shows molecule “A” which has a relatively high concentration and fast dynamics, whereas Panel D shows molecule “C”, which has a low concentration and slower dynamics.

NeuroRD is validated by comparison with an existing stochastic simulator (Smoldyn 2.05 [25] and deterministic solutions (XPPAUT 5.6.9 [38], Chemesis 2.1 [39]). The first validation evaluates NeuroRD simulations of diffusion of a single molecule species in a 10×11×1 µm rectangular cuboid subdivided into 110 subvolumes of size 1×1×1 µm. 2000 diffusing molecules (D = 300 µm^2^/s) are placed in the center of one edge of the slab (the source subvolume). The number of molecules in a given subvolume (or defined region in Smoldyn) reveals good agreement between Smoldyn and NeuroRD, both of which agree with the deterministic solution, illustrated in Fig. 3A for subvolumes at several distances from the source subvolume. The second validation set evaluates NeuroRD simulations of reactions alone in the same morphology as the first validation using two reversible bimolecular reactions (A+B↔C and A+C↔D). Although all four molecular species diffuse (required for reactions to proceed in Smoldyn), the molecules are distributed homogeneously in space so that there are no diffusional gradients. Fig. 3B shows that the time course and steady state values for Smoldyn and NeuroRD agree with each other and the deterministic solution. Note that the results for NeuroRD do not change if the molecules are made non-diffusible.

The next two validation sets evaluate NeuroRD simulations of reaction-diffusion systems, in which both the reactions and diffusion play a significant role in the dynamics. For the third validation, the same molecules, reactions and morphology described in the first validation set are used, but with different initial conditions. Molecules A, C, and D are initialized to zero, and 662 molecules of B are homogeneously distributed. After 100 ms, molecule A is injected in a single subvolume with a rate of 20 molecules/ms for 200 ms (total of 4000 molecules). Fig. 3C and D show the results for molecules A and C, respectively, in subvolumes at different distances from the source subvolume (where molecules are injected). Again, the time course for both stochastic simulators agree with each other and the deterministic solution. Fig. 3D further illustrates that the range of stochastic fluctuations are similar for both Smoldyn and NeuroRD. Though not illustrated, the reactions in Smoldyn are dependent on molecule proximity (approximate collisions), thus when reaction and diffusion rates produce a diffusion limited system, the Smoldyn solution departs from both NeuroRD and the deterministic solution. The ultimate validation compares stochastic results generated with NeuroRD to deterministic results generated with Chemesis for the full model which excludes the FRET sensor. Fig. S2A shows that mean cAMP concentration submembrane and cytosol in the stochastic simulation agree with the deterministic simulation results. Fig. S2B shows that a low concentration molecule in the submembrane region such as PKAc bound to PDE4B also agrees on average but shows large fluctuations that are not captured by the deterministic model.

The computational efficiency of NeuroRD is evaluated by comparison with Smoldyn for the above simulations, as well as simulations of larger numbers of molecules. Table 3 shows simulation time and memory allocated for the two stochastic simulators as a function of number of reactions, number of molecule species, and total number of molecules. NeuroRD simulations are between 2.5 and 1108 fold faster than Smoldyn. The limiting factor for speed in Smoldyn simulations is total number of molecules. Total number of molecules has little to no effect on NeuroRD speed, whereas the limiting factors in NeuroRD are the number of reactions or diffusing species, as well as the number of subvolumes. As described in the robustness section, simulation time of NeuroRD scales approximately linearly with number of subvolumes (Table 4).

**Table 3:** Comparison of scalability between NeuroRD and Smoldyn.

			NeuroRD		Smoldyn	
Simulation	# initial molecules	# injected	Time (h:mm:ss)	Memory (kb)	Time (h:mm:ss)	Memory (kb)
Diffusion	0	2000	0:00:02.86	1608	0:00:07.04	2344
Reaction	28853	0	0:00:05.97	1764	0:08:03.53	26524
Reaction & Diffusion I	662	4000	0:00:04.51	1764	0:02:48.90	22168
Reaction & Diffusion II	6619	40000	0:00:07.58	1772	2:19:58.00	23760

Time and memory allocation were measured for several sets of simulations (see section Validation in the text for details). All simulations were run for 3000 msecs and the total volume of the system was 110 µm^3^. The simulation *Diffusion* includes one molecular species and no reactions while all the remaining simulations have 4 molecular species and 2 reversible bimolecular reactions. The simulation labeled *Reaction* starts out of biochemical equilibrium albeit the distribution of molecules in space is homogeneous. *Reaction & Diffusion* (I and II), start in equilibrium but molecules are injected after 100 msecs disturbing both the homogeneous distributions of molecules and their biochemical equilibrium. Concentrations in simulation *Reaction & Diffusion II* are well within the physiological range (highest molecular species (A) concentration: ∼400 nM).

**Table 4:** Scalability of NeuroRD as a function of mesh size (space discretization) and time step.

			Time (h:mm:ss)	Memory (kb)
Δt (msec)	Δx (µm)	# Subvolumes		
0.1	0.9333	60	0:47:10.98	128744
0.05	0.451	248	5:57:06	190704
0.015	0.229	976	63:19:53	311644

The simulation of cAMP microdomains produced by PKA activation of PDE4s (without H30, as plotted in Figure S5) was run with different mesh sizes and timesteps. The simulation time and amount of memory allocated shows that *NeuroRD* scales approximately linearly with number of subvolumes, and number of timesteps.

### Simulations demonstrates that cAMP microdomains are not due to physical barriers

Recent experimental data collected using an Epac-based FRET sensor (H30) shows a distinctive submembrane cAMP microdomain in HEK293 cells due to differential PDE activity and location [16]; however, others suggest that this microdomain is the result of impeded cAMP diffusion [10]–[13]. The two most prominent PDE4 sub-families found in HEK293 cells are PDE4B, which is anchored at the submembrane, and PDE4D, which is found in the cytosol [16]. To test the role of compartmentalized PDE4s as opposed to impeded cAMP diffusion in producing microdomains, a computational model of cAMP production, PKA activation and compartmentalized PDE activity is developed using the *NeuroRD* software.

In order to ensure rigorous quantitative comparison between experimental and modeling results, simulations replicate the original experimental protocols [16] and include cAMP binding to the FRET sensor. Thus, the submembrane cAMP microdomain is calculated from a simulation in which the Epac-based H30 (which binds a single molecule of cAMP) is included as a submembrane-anchored protein (analogous to measuring submembrane cAMP from cell cultures in which the membrane-bound H30 is expressed). The cytosolic cAMP concentration is calculated from a simulation in which H30 is included as a cytosolic protein (analogous to measuring cytosolic cAMP from cell cultures in which the cytoplasmic H30 is expressed). The simulated FRET is calculated from concentrations of free H30 and cAMP-bound-H30 and includes contamination terms constrained by experimental measurements (Eq. 7 and 8). Fig. 2 shows good agreement between simulated and experimental FRET calibration data, both in dose-response and time course regimes, in this case where the experimental cAMP concentration is controlled by loading the HEK cell with a known amount of cAMP from a patch pipette. This agreement in the simulated and experimental FRET signal suggests that the model simulations will correctly predict cAMP spatio-temporal dynamics underlying the experimental FRET signal under conditions of agonist application.

**Figure 4:**
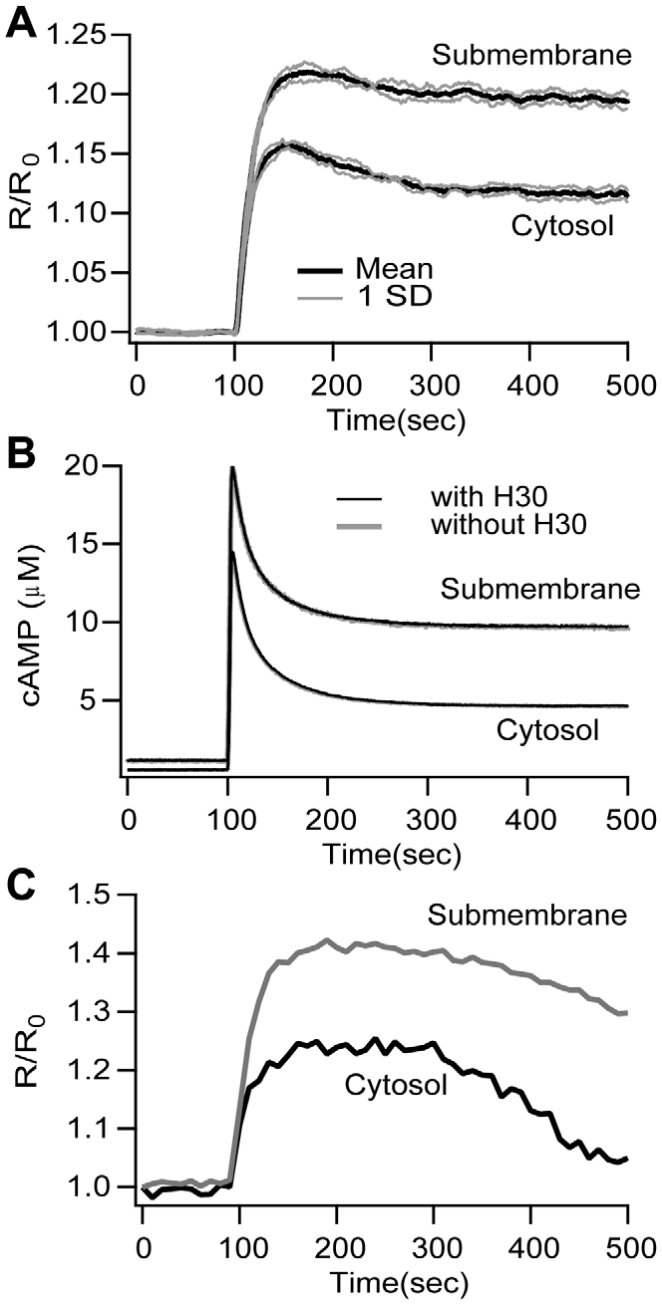
The theoretical FRET signal and cAMP concentration show microdomains without diffusional barriers. (A) The FRET signal for the submembrane region is 6.8% higher than the cytosol. Mean (black traces) and ±SD (gray traces, n = 5). (B) Difference between submembrane and cytosolic cAMP concentration is similar to that observed for the FRET signal, and is independent of overexpression of the H30 sensor. The model cell with H30 is shown in black; the model cell without H30 is shown in gray. SD traces are not illustrated because they overlap with the mean. No diffusional barriers are present for these simulations. The expression of the sensor does not disturb the cAMP microdomain, therefore the difference between submembrane FRET and cytosolic FRET is not an artifact of the method. (C) Representative kinetics of FRET changes recorded in cells expressing either the membrane targeted sensor mpH30 or the cytosolic sensor H30 [16], [56] upon stimulation with 1µM PGE_1_. FRET experiments were performed as described previously in [16].

The first set of simulations evaluates whether diffusional barriers are required for the cAMP microdomain as measured by FRET. This first step aims at reproducing the results previously reported [16]. Fig. 4A shows that the model successfully reproduces the experimental FRET signal, including the difference in cAMP concentration between submembrane and cytosolic compartments. Comparison between the experimentally calculated FRET signal (Fig. 4C) and the theoretically derived FRET signal shows that both traces have a sharp increase (rising phase) right after stimulation is delivered at 100 s. Likewise, FRET peak value is reached ∼100 s after stimulation has started in both experiment and simulation. Because this simulation uses experimentally constrained values for diffusion, this result confirms the experimental result that the cAMP microdomain does not require diffusional barriers.

The simulated cAMP traces can be regarded as a prediction of the cAMP values for these experimental protocols because simulations of both the cAMP and the FRET match the experimental data for the calibration protocol. Accordingly, Fig. 4B shows the cAMP signal underlying the FRET signal shown in Fig. 4A. The cAMP concentration is higher in the submembrane region compared to the cytosol as expected from the FRET signal. Nonetheless, the fast cAMP signal dynamics do not appear in FRET traces because the rate constant for H30 binding to cAMP is not fast enough to capture the fast transient in the cAMP signal, which is apparent by comparing the time course of cAMP with that of the FRET signal.

An advantage of simulations is the ability to evaluate the cAMP microdomain in a single model cell without expression of the FRET sensor. Thus, the second step repeated the simulation in a model which lacked H30, to confirm that the observed microdomain is not a result of disruption of normal cellular signaling or unbalanced FRET sensor expression. Fig. 4B shows that the cAMP microdomain occurs in a single cell without H30 expression. The spatio-temporal profile of cAMP concentration for this case is further illustrated in Fig. S5B and in the Movie S1. The difference between cAMP submembrane concentration and cAMP cytosolic concentration is identical to the case with the sensor. The simulation showing that H30 expression does not affect the cAMP microdomain further reinforces the previous results and confirms the experimentalists' assertion that the cAMP microdomain is not an artifact of either H30 expression or unbalanced concentrations of the FRET sensor.

### PDE4s: molecular mechanisms responsible for cAMP microdomains

Although these computational experiments show that the cAMP microdomain can be reproduced without diffusional barriers, the identification of the exact mechanism responsible for the compartmentalization requires additional simulations. Experiments by Terrin et al. show that silencing the cytosolic PDE4D creates a low concentration, submembrane cAMP microdomain whereas silencing the submembrane PDE4B does not change the cAMP microdomain [16]. To further evaluate the role of PDE localization and subtypes, the second set of simulations replicate the experiments by simulating selective silencing of specific PDE4s. To approximate experiments where PDE4s were selectively silenced, the appropriate PDE4 concentration is lowered to 10% of its control value. In addition, the stimulation is lowered to 1.3% of its original value for the silencing of PDE4D and to 65% of its original value for silencing of PDE4B in order to yield comparable cAMP peak concentrations. These lower stimulation values approximate a compensatory down-regulation of adenylyl cyclase, which could explain the weaker FRET signal observed experimentally [16], and also prevent cAMP from reaching unphysiological levels after stimulation. Because lowered PDE produces a change in cAMP basal concentration, the simulation is re-equilibrated before applying the stimulation, analogous to the re-equilibration of culture cells after transfection, while waiting for expression of siRNA.

**Figure 5:**
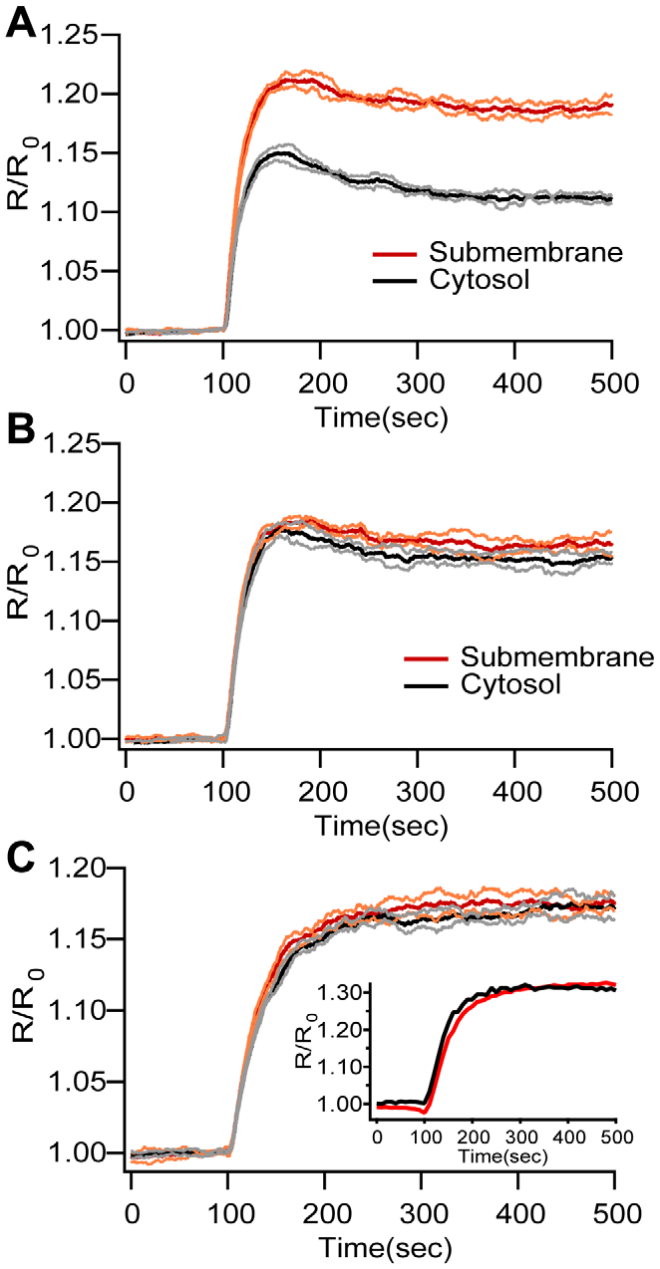
Mechanisms underlying cAMP microdomains. (A) Silencing of PDE4B does not eliminate the submembrane microdomain. (B) Silencing of PDE4D does eliminate the submembrane microdomain. (C) Blocking PDE4 phosphorylation by PKAc eliminates the submembrane microdomain, and also eliminates the decay of the FRET signal from the peak. There is substantial overlap of cytosol and submembrane standard deviation traces. These results suggest that PKA is the main effector of the microdomain through phosphorylation of PDE4s (inset shows representative experimental data). Mean and ±SD traces in red and orange for submembrane and black and gray for cytosol, respectively (n = 5).

Simulation results agree with experiments in regard to assigning distinctive roles to specific PDE subfamilies in cAMP microdomain generation. Simulation of PDE4B silencing does not eliminate the cAMP microdomain: simulated FRET is higher in the submembrane region than in the cytosol (Fig. 5A). In contrast, simulation of PDE4D silencing eliminates the microdomain: the FRET signals in cytosol and submembrane regions are comparable (Fig. 5B). Simulated PDE4D silencing does not produce a low concentration, submembrane cAMP domain (as observed experimentally), suggesting that other mechanisms might be responsible for this particular result (see discussion).

### cAMP microdomain requires PKA phosphorylation of PDE4s

PKA may play a role in generating the cAMP microdomain because PDE4 activity is enhanced by PKA phosphorylation [16], [40]. Indeed, H89 (a PKA inhibitor) eliminates the cAMP microdomain measured with the FRET sensor [16]. The third computational experiment replicates this PKA inhibition experiment and further asks if PKA phosphorylation of PDE4s alone is sufficient to explain the microdomain. If other PKA targets are essential for the microdomain, simulated block of PKA phosphorylation will not eliminate the microdomain because the simulation described here does not include these other PKA targets.

Blocking the phosphorylation of PDE4s by PKA catalytic subunit takes the model out of its original equilibrium and therefore, before applying the stimulation, the ten min of simulation time allows the system to re-equilibrate, analogous to the application of H89 ten min prior to imaging as performed by Terrin et al. [16]. Adenylyl cyclase activity is stimulated using an amplitude that is ten times smaller than the control case, again mirroring experiments.

Blocking of PKA phosphorylation of PDE4s eliminates the microdomain: the FRET signal at the submembrane and cytosol are comparable (Fig. 5C). This suggests that PKA phosphorylation of PDE4s is necessary and sufficient to implement the microdomain as measured by FRET imaging. Furthermore, these simulations capture another characteristic of the system: the decay from peak FRET signal is abolished. The absence of decay when PKA is blocked in both experiments and simulations shows that at least part of the decay kinetics is due to PKA phosphorylation of PDEs. In order to further evaluate this hypothesis, the amount of PKA is increased by a factor of four to simulate experimental conditions using a PKA-based FRET sensor. The increased PKA enhances the decay in the cAMP trace as compared to control (Fig. S3), in agreement with experimental data [16]. In summary, the model reproduces the effects of PKA quantity on the decay of the FRET signal, confirming the role of PKA phosphorylation of PDE on cAMP dynamics. Nonetheless, other mechanisms not included in the simulation may be contributing since the magnitude of the decay observed in control simulations is smaller than that observed in experiments.

### Propagation of cAMP microdomains to downstream targets

One function of cAMP microdomains is to achieve localized activation of targets such as PKA. The PKA holoenzyme is anchored and does not diffuse, but after cAMP binds to the regulatory subunit, the catalytic subunit is released, diffuses throughout the cell and phosphorylates various targets including PDE4s. Therefore, propagation of the cAMP microdomain is examined by evaluating cAMP-bound-PKA, PKA catalytic subunit, and phosphoPDE4s. The PKA holoenzyme concentration is higher in the submembrane than in the cytosol, thus we also examine the fraction of PKA bound to cAMP (cAMP-bound-PKA divided by the total PKA).

**Figure 6:**
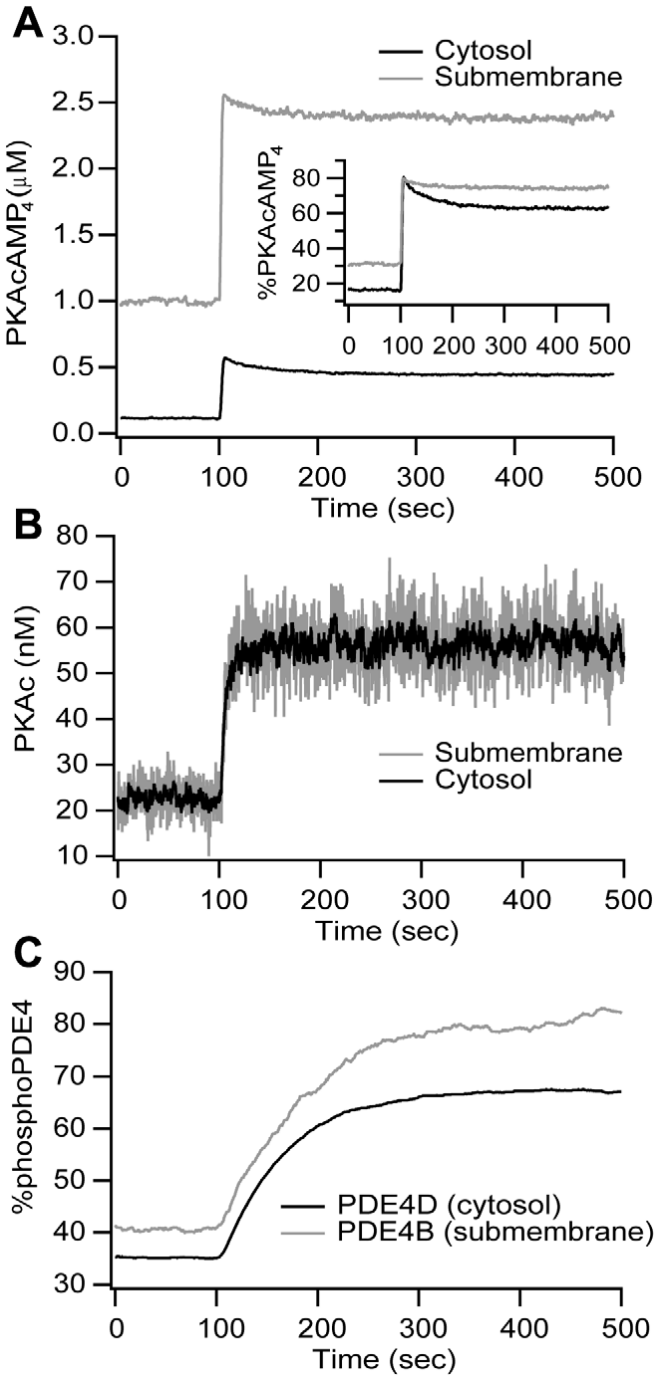
Propagation of cAMP microdomains to downstream targets. (A) The increase in the quantity of PKA with 4 cAMP molecules bound is greater in the submembrane region than in the cytosol. However, the percent increase is the same submembrane and cytosol. (B) PKA catalytic subunit (PKAc) concentration is the same in submembrane and cytosolic compartments. (C) The higher cAMP concentration observed submembrane translates into a larger fraction of phosphorylated PDE4s in the submembrane (pPDE4B) as compared to the cytosol (pPDE4D). A single representative trace is illustrated in each panel.

Fig. 6A shows that the increase in the quantity of PKA with 4 cAMP molecules bound is greater in the submembrane region than in the cytosol; however, the percent increase is the same submembrane and cytosol. The reason for the discrepancy between total increase and percent increase is that the initial percentage of fully bound PKA is higher submembrane than in the cytosol, because initial submembrane cAMP concentration is greater than the affinity of cAMP for PKA. Fig. 6B also shows the quantity of free PKA catalytic subunit. The concentration in the submembrane region equals that in the cytosol (Fig. 6B), both the initial value and after stimulation. Diffusion of the PKA catalytic subunit is not likely to explain the lack of a PKA microdomain because the diffusion constant of the PKA catalytic subunit is ten times smaller than that for cAMP. Instead, these results reinforce the importance of degradative mechanisms (e.g. PDE4s) for the production of microdomains: no microdomain of PKA catalytic subunit is observed because the model does not include mechanisms that directly consume the PKA catalytic subunit, as opposed to the situation with cAMP.

The quantity of the free PKA catalytic subunit may not accurately reflect propagation of the microdomain to PKA targets. Due to the large quantities of PDE4D, most of the PKA catalytic subunit is not free, but is bound to PDE4D. Thus, Fig. 6C plots percentage of phosphorylated PDE4 to evaluate whether a microdomain of PKA *activity* is apparent. Fig. 6C shows that the phosphorylation of the membrane-bound PDE4B is higher and increases more than the activity of the cytosolic PDE4D, suggesting that the microdomain propagates downstream.

### Diffusion plays a minor role in generation of the cAMP microdomains

**Figure 7:**
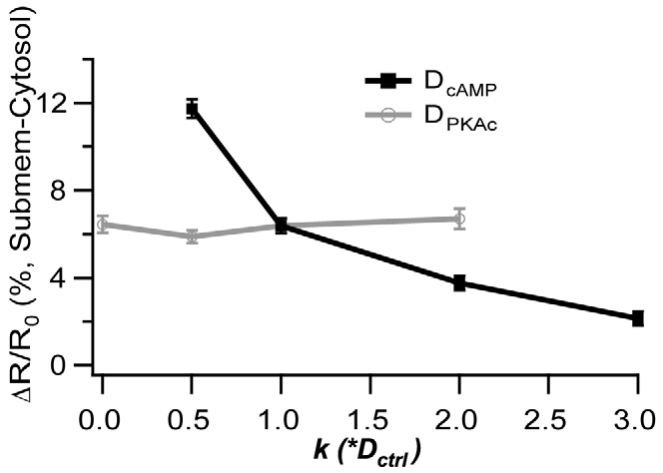
Amplitude of the microdomain is influenced by cAMP diffusion coefficient, but not by PKAc diffusion coefficient. Impeded diffusion of cAMP is not required for the microdomain, but influences the concentration difference between submembrane and cytosol. The faster the cAMP diffusion coefficient, the smaller the difference between submembrane and cytosol concentration (measured as difference between FRET ΔF/F submembrane and FRET ΔF/F cystosol (solid black line, black squares). cAMP diffusion coefficient ranges from k = 0.5 to k = 3 times its control value of 295 µm^2^/s). Diffusion of the PKA catalytic subunit plays no significant role in generating cAMP microdomains (solid gray line, open squares). PKAc diffusion constant ranges from k = 0 to k = 2 times its control value of 59.54 µm^2^/s.

Although these simulations confirm that PDE4s play the main role in controlling cAMP microdomains, diffusion may still play a role because an infinitely fast diffusion constant theoretically would produce a well stirred and homogenous distribution of molecules. To delineate the role of cAMP diffusion and to evaluate the robustness of the model to parameter variations, simulations are repeated with the cAMP diffusion constant ranging from one half to three times its control value, representing the range of experimentally measured values. Simulations show that reducing the speed of cAMP diffusion increases the concentration difference between submembrane and cytosol, while increasing the speed of cAMP diffusion diminishes, but does not eliminate, the cAMP concentration difference (Fig. 7). Thus, the results are not dependent on the precise value chosen for the cAMP diffusion constant. PKA is another important and diffusible molecule in the model; thus, the effect of diffusion of the PKA catalytic subunit (PKAc) also is evaluated, by repeating simulations with the PKAc diffusion constant ranging from one half to two times its control value. Fig. 7 shows that the change in the PKAc diffusion constant produces no change in the magnitude of the cAMP concentration difference, even in the most extreme case with no PKAc diffusion. Though diffusion of the PKA catalytic subunit is slower than cAMP, PKAc diffusion is fast compared to its inactivation (rebinding to the regulatory subunit) so that PKAc diffuses to the cytosol to phosphorylate PDE4D, thereby generating the cAMP microdomain. In summary, the cAMP microdomain does not require impeded diffusion, but the extent of the cAMP concentration difference is affected by the diffusion constant of cAMP, though not that of PKAc.

### Robustness to Parameter Variation

To further explore the sensitivity of results to parameter variations, simulations are repeated with different values of the least constrained parameters, such as the quantities of AC and PDE4; these are reduced jointly to maintain the same basal cAMP concentration. Simulations show that the size of the cAMP microdomain is robust to changes in AC and PDE4 quantities (Fig. S4). Thus, overall, the results are robust to changes in the quantity of AC, PDE4, and PKA (Fig. S3), as well as the diffusion rate of PKAc and cAMP (Fig. 7). Simulations also are repeated using two different and smaller mesh sizes (with increased numbers of subvolumes to maintain the same total simulated volume). Fig. S5 shows that the difference in cAMP concentration between submembrane and cytosolic regions is robust to changes in the mesh size. In addition, simulations with a larger, 10×15 subvolume 2-dimensional grid (extending the cell in the direction parallel to the membrane, with subvolumes of same size as defined before, 0.93×0.93×0.5 µm), representing the entire 2-dimensional projection of the 3-dimensional cell, yield similar results (data not shown).

## Discussion

The stochastic simulations described here explored the roles of diffusion, PKA and PDE4s in generating spatial microdomains. The HEK293 cell model included cAMP production, degradation by PDE4s, and the main cAMP effector PKA. In order to precisely compare the simulation results with experiments, the model also included the H30 sensor in either the cytosol or the submembrane regions. A theoretical FRET equation was derived and its parameters were constrained by experimental measures which allowed the calculation of a FRET signal based on the concentrations of unbound H30 and cAMP-bound-H30. The simulations not only replicated experimental results, but also provided further tests of the mechanisms underlying cellular microdomains that would be difficult using current experimental methods and preparations.

Control simulations quantitatively reproduce the cAMP microdomain as measured by the FRET signal. Simulations without H30 expression, which compare submembrane and cytosol cAMP within a single cell, yield similar results to control simulations, demonstrating that the cAMP microdomain is not an artifact resulting from either disruption of the cellular signaling or unbalanced FRET sensor expression. Various characteristics of the simulated and experimental FRET signals are in good agreement: rising phase, peak value, and difference between submembrane and cytosol. As expected, the simulated cAMP concentration itself has a different time course than the FRET signal as a result of the slow rate of cAMP binding to H30. Thus, measures of cAMP using FRET are likely to underestimate peak cAMP concentrations. An alternative technique such as genetically encoded cyclic nucleotide-gated channels [41] provides high temporal resolution, but can only measure submembrane cAMP; thus, the use of cyclic nucleotide-gated channels is not a viable approach for measuring cAMP microdomains.

### Dynamics of the cAMP signal

Simulations and experimental data diverge after the initial rising phase and peak. Specifically, the simulated FRET signal has a modest decay (Fig. 4A) while the experimental FRET trace has a pronounced decrease (Fig. 4C). This divergence might be explained by different mechanisms that are beyond the scope of this study. First, the model presented here does not explicitly include the G protein-coupled receptor (GPCR) and its production of active G_α_GTP. Rather, it approximates GPCR activation and desensitization by injecting a quantity of G_α_GTP over a short duration. Desensitization of GPCRs through either receptor inactivation or internalization is a limiting factor in cAMP production and is mediated by PKA, GRK and β-arrestin [41], [42], [40]. Second, the model does not include degradation of G_α_GTP which also may contribute to the pronounced decay observed in the FRET signal. Third, additional PDE-independent mechanisms related to cAMP removal [42] not included in the model might be responsible for the temporal signature observed in FRET experiments. Though none of these mechanisms are included, control simulations still show moderate decay of the FRET signal, which is abolished in simulations when PKA activity is blocked. Thus, one of the mechanisms contributing to the decay phase is PKA phosphorylation of PDE4s, because inhibition of PKA activity, both simulated and in experiments, produces a decrease in the decay of the FRET signal (Fig. 5C). The decay rate is important because the time course of cAMP, whose decay is controlled by PDEs, strongly influences the spatial extent of the cAMP signal by limiting the time available for diffusion [43].

### The Role of Phosphodiesterases in Producing cAMP Microdomains

Several simulations confirm the hypothesis that PDE4D is the main mechanism responsible for the cAMP microdomain, and that impeded diffusion or physical barriers are not required. First, the microdomain does not require a lowered diffusion coefficient, but is robust to changes in diffusion constants of cAMP and PKA catalytic subunit (Fig. 7). Second, simulated silencing of PDE4D disrupts the cAMP microdomain, whereas simulated silencing of 4B does not. Thus, in both the simulations and experiments, PDE4D acts as a sink, lowering cAMP concentration in the cytosol more so than in the submembrane compartment [3], [15], [16].

One specific experimental result could not be replicated: while experimental silencing of PDE4D results in a microdomain of low cAMP concentration in the submembrane region, simulated silencing of PDE4D abolishes the high concentration microdomain but does not produce a low concentration microdomain in the submembrane region. Though the mechanisms responsible for this particular experimental observation are yet to be fully explained, additional simulations and theoretical considerations suggest that the mere absence of the PDE4D degradative mechanism from the cytosol is not sufficient to move the highest concentration region away from its source. An active mechanism is required to move cAMP from its submembrane site of production to the cytosol. One potential mechanism is similar to the pumps which maintain the potassium concentration higher inside a cell, but this is implausible given the absence of a membrane separating the two compartments. A more likely explanation for the lower cAMP concentration in the submembrane region as compared to the cytosol is that the main source of cAMP shifts from the membrane to the cytosol. Recent experimental results suggest that, contrary to previous assumptions, GPCRs remain active after internalization, continuing to stimulate cAMP production in association with internalized AC [44], [45]. A relative increase in cytosolic cyclase activity has been postulated to occur in response to PGE1 stimulation of cardiac myocytes [46], [47]. An experiment to test this idea requires selectively blocking cyclases in the cytosol, but not the submembrane region, combined with silencing of PDE4D.

The importance of phosphorylation of PDE4 in the HEK293 cell extends the results of Neves et al. [22] to cells with minimal diffusional barriers. Neves et al. [22] investigate microdomains in neurons using a deterministic simulation (including regions representing dendrites and soma), and demonstrate that PDE4s contribute to cAMP microdomains that develop in dendrites, as compared to the soma. Two different mechanisms underlie their cAMP microdomain: (1) the surface to volume ratio, which is higher in long thin dendrites (100 µm length by 1 µm diameter) than in the round soma (20 µm diameter), and (2) the diffusional barrier created by the small diameter of the dendrite. Consistent with these two mechanisms, increasing dendritic diameter to 3 µm eliminates the difference between soma and dendrite cAMP concentration. In both models, an additional contribution to the magnitude of microdomains is the speed of diffusion, though this is more important in longer structures such as neuronal dendrites (as opposed to HEK293 cells). Neves et al. do not explore the role of different PDE4 subtypes, with their specific subcellular locations, whereas the present research demonstrates that the location of two types of PDE4s (and the regulation of their activities by PKA) produces a gradient of cAMP orthogonal to the membrane. In neurons, local synaptic activation, together with diffusional barriers and degradative mechanisms, will enhance the formation of microdomains, which are important for information processing. Thus one prediction of this model, not explored by Neves et al., is that PKA phosphorylation of PDE4s contributes to synaptic specificity.

Propagation of cAMP microdomains to downstream targets is observed in these simulations, similar to other experimental results (e.g. [22], [48]). The increase in the quantity of cAMP-bound-PKA is greater in the submembrane region than in the cytosol. Although the quantity of free PKA catalytic subunit does not reflect the cAMP microdomain, the increase in phosphorylation of PDE is greater in the submembrane region than in the cytosol. Nonetheless, the downstream submembrane microdomain exhibits a smaller difference between submembrane region and cytosol, due to the morphology of the cell and the basal cAMP concentration. In the cytosol the basal cAMP concentration is near the K_D_ for PKA binding to cAMP, but in the submembrane region the basal cAMP concentration is higher than the K_D_. This implies that increments in cAMP are translated into smaller increments of cAMP-bound-PKA in the submembrane region as compared to the cytosol. Thus, if the basal cAMP concentration were lower in both regions, as has been observed in other cell types [49], the cAMP microdomains would have propagated more strongly to downstream targets such as PDE4. The morphology of the cell is relevant because the diffusion constant relative to the cell size (or dendrite length) contributes to the amplitude of the microdomain. Neves et al. finds that propagation of the dendritic cAMP microdomain to downstream PKA and MAPK (Mitogen-Activated Protein Kinase) is decreased when the radius of the dendrite was increased [22].

### NeuroRD: a new tool for stochastic simulations of reaction-diffusion systems

One important aspect of our model is its implementation using *NeuroRD*, the computationally efficient, stochastic (Monte Carlo) reaction-diffusion software. The computational efficiency of the algorithm allows for simulating a relatively large cell, such as the HEK293 cell, subdivided into small subvolumes, in which the small numbers of molecules implies that reaction and diffusion will occur randomly. In the highly non-linear and complex reaction-diffusion systems of cells, accurate diffusion requires either tracking individual molecules, e.g. MCell [33] or Smoldyn [25], or subdivision into sufficiently small subvolumes. [50]. The large numbers of molecules in a large volume makes tracking individual molecules or exact stochastic simulation (e.g. [32], [51], [52]) computationally expensive, and possibly prohibitive, as demonstrated by the comparison between Smoldyn and NeuroRD (Table 4). As the numbers of molecules increases (without changing the total volume and still remaining in the physiological range), the computational advantage of NeuroRD increases.

NeuroRD has similarities and differences with the MesoRD software [52], which uses the “spatial next” algorithm. It is similar in that MesoRD subdivides space into subvolumes to avoid tracking individual molecules. The spatial next algorithm used by MesoRD extends the next reaction method [24] by including diffusion to adjacent subvolume as a possible reaction event. NeuroRD differs from MesoRD in that NeuroRD is a spatial extension of Gillespie's *tau-leap* algorithm [53], which allows multiple reaction events at each time step, instead of a single reaction event. Thus, NeuroRD allows multiple reaction and diffusion events at each time step. Additional efficiency is achieved with a table lookup for Binomial random numbers [23].

The ability of NeuroRD to implement a stochastic, large scale simulation is revealed by the production of cAMP microdomains in the HEK293 cell. NeuroRD is utilized to account for the stochastic behavior of the small number of PKA catalytic subunits. Incorporating spatial aspects of signaling pathways becomes critical as experiments provide more information on the importance of subcellular location of molecules. The computational efficiency of spatial, stochastic simulations with NeuroRD makes this software ideal for simulation of neurons, which have numerous small compartments (spines) attached to relatively large compartments (dendrites). Imaging experiments show that microdomains of calcium occur in spines, and this software would be ideal for exploring mechanisms that produce microdomains in dendritic spines and neuronal dendrites [54], [55].

## Supporting Information

Figure S1 Flowchart illustrating the reaction-diffusion algorithm used by NeuroRD. (A) At each timestep, the number of diffusing molecules is calculated first, then the number of molecules in each subvolume is updated, then the number of reaction events is calculated, and last the number of molecules in each subvolume is updated again. (B) Algorithm used to choose the number of molecules either reacting or diffusing: small populations (<120) use lookup tables while larger population use Poisson tables (if Np<20) or the Gaussian distribution (if Np>20). (C) Algorithm used to choose the destination of diffusing molecules among neighboring subvolumes: If the number of diffusing molecules, k, is smaller than 4 times the number of neighboring subvolumes, then the destination subvolume of each particle is determined randomly (independently). Otherwise, the number of particles, m, diffusing to a subvolume is calculated from the binomial distribution, where the probability of diffusing to a particular subvolume, p_c_, is the ratio of p_m_ (calculated from Eqn 2) for that subvolume to the total of p_m_ for all adjacent subvolumes.
(1.57 MB EPS)

Figure S2 Comparison of HEK293 cell model simulated in NeuroRD and Chemesis. (A) cAMP traces in cytosol and submembrane regions in deterministic and stochastic simulations overlap. (B) The average value of low concentration species, such as PKAc-PDE4B and PKAc-PDE4B-cAMP located in the submembrane region, show excellent agreement between deterministic and stochastic simulations, but the large fluctuations in molecule quantities are not captured by the deterministic model.
(0.69 MB EPS)

Figure S3 Increased PKA enhances the decay in the cAMP trace as compared to control. Comparison of cAMP traces generated in simulations with control parameters and with PKA quantity increased by a factor of four. Increased PKA makes the decay steeper. PDE dephosphorylation rate is three times faster in these simulations in order to maintain similar basal levels to control simulations.
(0.35 MB EPS)

Figure S4 Model is robust to decreases in quantities of AC and PDE4s. Bar plot shows that the difference between submembrane and cytosol cAMP concentration at basal and peak are similar for Control and Reduced AC and PDE4s simulations. Basal values are shown by the bars and axis on the left, and peak values shown by the bars and axis on the right. AC and both PDE4s are scaled by the same factor. Stimulation is adjusted in order to produced similar peak cytosol amplitude.
(0.10 MB EPS)

Figure S5 Model results are robust to changes in mesh size. (A) Simulations with Δx = 0.933 µm, 0.456 µm, 0.229 µm result in virtually equal cAMP microdomain sizes. Therefore the size of the subvolumes does not affect the size of the cAMP concentration difference between submembrane and cytosol compartments. (B) Snapshots show cAMP spatial profile of the modeled system at different points in time (⊗ 20 secs, * 110 secs and ⊕ 500 secs) for the simulation with Δx = 0.933 Δµm shown in A.
(0.35 MB EPS)

Movie S1 cAMP Spatio-temporal profile for the simulation in Fig. S5. Movie illustrates rapid development of the high cAMP concentration at the submembrane and the persistence of the low concentration in the center of the cell slice modeled. There are no concentration gradients along the membrane; all concentration gradients are orthogonal to the membrane, justifying averaging over these subvolumes to produce the submembrane traces.
(0.57 MB MPG)
